# Fabrication of Stromal Cell-Derived Factor-1 Contained in Gelatin/Hyaluronate Copolymer Mixed with Hydroxyapatite for Use in Traumatic Bone Defects

**DOI:** 10.3390/mi12070822

**Published:** 2021-07-14

**Authors:** Yun-Liang Chang, Chia-Ying Hsieh, Chao-Yuan Yeh, Chih-Hao Chang, Feng-Huei Lin

**Affiliations:** 1 Department of Biomedical Engineering, National Taiwan University, No. 1, Sec. 1, Jen-Ai Road, Taipei City 10051, Taiwan; knifex@gmail.com (Y.-L.C.); larissa19950718@gmail.com (C.-Y.H.); 2 Department of Orthopaedic Surgery, National Taiwan University Hospital, No. 7, Chung Shan South Road, Taipei City 10002, Taiwan; 3 Integrative Stem Cell Center, China Medical University, No. 2, Yude Road, Taichung City 40447, Taiwan; joeyeh@gmail.com

**Keywords:** tissue engineering, biomaterial, hyaluronate, mesenchymal stem cells, hydroxyapatite, bone regeneration, gelatin, bone defect

## Abstract

Bone defects of orthopedic trauma remain a challenge in clinical practice. Regarding bone void fillers, besides the well-known osteoconductivity of most bone substitutes, osteoinductivity has also been gaining attention in recent years. It is known that stromal cell-derived factor-1 (SDF-1) can recruit mesenchymal stem cells (MSCs) in certain circumstances, which may also play an important role in bone regeneration. In this study, we fabricated a gelatin/hyaluronate (Gel/HA) copolymer mixed with hydroxyapatite (HAP) and SDF-1 to try and enhance bone regeneration in a bone defect model. After material characterization, these Gel/HA–HAP and Gel/HA–HAP–SDF-1 composites were tested for their biocompatibility and ability to recruit MSCs in vitro. A femoral condyle bone defect model of rats was used for in vivo studies. For the assessment of bone healing, micro-CT analysis, second harmonic generation (SHG) imaging, and histology studies were performed. As a result, the Gel/HA–HAP composites showed no systemic toxicity to rats. Gel/HA–HAP composite groups both showed better bone generation compared with the control group in an animal study, and the composite with the SDF-1 group even showed a trend of faster bone growth compared with the composite without SDF-1 group. In conclusion, in the management of traumatic bone defects, Gel/HA–HAP–SDF-1 composites can be a feasible material for use as bone void fillers.

## 1. Introduction

Bone defects refer to the destruction of bone integrity, and they remain a challenge in clinical practice [1,2]. Large bone defects over a critical size may not only cause difficulties in fracture reduction and fixation, but may also compromise bone healing and eventually lead to the delayed union or nonunion of bone [3]. When treating large bone defects, most surgeons will try to fill certain kinds of bone grafts that incorporate internal fixation to facilitate indirect bone healing [4,5,6]. Various ceramics, bioglasses, or even metals have been introduced for bone regeneration [7,8]. In order to overcome the limitations of current commercial bone grafts, many researchers have focused on tissue engineering, especially naturally derived or synthetic materials, hoping that bone healing may occur spontaneously [9,10,11]. Tissue engineering involves a combination of material science and cell biology to provide brand new biomaterials that can mimic real tissues and stimulate in vivo regeneration, or can be developed in vitro into a functional tissue before their implantation. Therefore, the possible ways to promote bone healing include using materials that incorporate bone healing factors or materials that seed bone-derived cells.

Gelatin (Gel) is denatured collagen. It has been widely used in biomaterials, including tissue engineering and drug delivery systems [12,13,14,15]. Gelatin has good biocompatibility, biodegradability, and has a low cost. It also enhances cell attachment, differentiation, and proliferation. Recently, gelatin-related scaffolds have been popularized in bone tissue engineering [16]. Integrated with 3D printing and various coatings, gelatin scaffolds can be applied into many different needs. Hyaluronate (HA), a nonsulfated glycosaminoglycan, is an essential component of the extracellular matrix. It is biodegradable, biocompatible, and is involved in cell–cell interactions, cell–matrix adhesion, and cell motility [17,18,19]. In order to increase the retention time of the prepared composite, 1,4-butanediol diglycidyl ether (BDDE) serves as a crosslinker. It is stable and biodegradable, and its degraded products are nontoxic [20]. In order to increase the mechanical strength, hydroxyapatite is added. Hydroxyapatite (HAP) is one of the components of bone, and it has good biocompatibility and an osteoconductive nature [21]. HAP-based composite scaffolds are already widely used in bone tissue regeneration [22]. Additive manufacturing methods for this kind of HAP-based scaffold even provide greater potential in tissue engineering due to higher customization ability [23]. Stromal cell-derived factor-1 is also known as C-X-C motif chemokine 12 (CXCL12), which is expressed in many tissues including bone marrow. It can also be induced in an injured periosteum. SDF-1 may be a potential bone healing factor because it has been found to be capable of recruiting mesenchymal stem cells toward defect sites. As a result, SDF-1 can be osteoinductive during bone growth and even enhance the bone regeneration process [24,25,26].

In this study, a Gel/HA copolymer was fabricated at a ratio of 85:15 (Gel:HA = 85:15), crosslinked by BDDE, and mixed with HAP at a ratio of 25:75 (Gel/HA:HAP = 25:75) [27]. The material properties were tested by XRD, FTIR, swelling ratio, degradation rate, and insulin release profile. In the in vitro study, a WST-1 assay for biocompatibility and an MSC recruitment test for SDF-1 chemotaxis of mesenchymal stem cells were performed using the cbMSC-hTERT cell line. In the in vivo study, surgery involved three groups: the control group, the Gel/HA–HAP–SDF-1 composite group, and the Gel/HA–HAP composite group. Micro-CT was used to analyze how well bone grew, H&E stains were used to check the histology, Masson trichrome stains were used to assess new bone formation, and blood tests were used to confirm systemic nontoxicity. We hypothesized that this Gel/HA–HAP–SDF-1 composite might show a certain degree of osteoinductivity, which can enhance the bone regeneration process in animal models of traumatic bone defects.

## 2. Materials and Methods

### 2.1. Basic Materials

Materials including hyaluronic acid sodium salt (HA), gelatin type A from porcine skin (Gel), 1,4-butandiol diglycidyl ether (BDDE), and calcium hydroxide were purchased from Sigma-Aldrich (St. Louis, MO, USA). On the other hand, cbMSC-hTERT cells were acquired from the American Type Culture Collection (ATCC). Stromal cell-derived factor-1 (SDF-1) was bought from PeproTech (Cranbury, NJ, USA).

### 2.2. Synthesis of Hydroxyapatite

Dropwise 0.3 mole/L orthophosphoric acid (H_3_PO_4_) was added to 0.5 mole/L calcium hydroxide (Ca(OH)_2_) solution at a rate of three milliliters per minute. The starting volume of the calcium hydroxide solution was 100 mL. In order to reach a Ca/P molar ratio of 1.67, approximately 0.17 mL of 0.3 mole/L phosphoric acid was added. The pH value of the mixture was then adjusted to 8.5. The mixture was stirred at 85 °C for 2 h. After that, it was left standing for 24 h at 85 °C. The precipitated powder was collected and washed with double-distilled water three times before being freeze-dried [28,29].

### 2.3. Synthesis of Gel/HA–HAP–SDF-1 Composite

HA (0.5 wt.%) and Gel (10 wt.%, 300 Bloom) were dissolved in distilled water above 37 °C. HA and Gel solutions were mixed at a volume ratio of 85:15, and BDDE was added as a crosslinker to the mixture at a concentration of 0.5 vol%. The mixture was then mixed with a magnetic stirrer at 37 °C for 24 h. HAP and the organic materials (Gel and HA) were mixed at a weight ratio of 75:25. When the temperature of the mixture achieved room temperature, one hundred nanograms per milliliter of SDF-1 was added. The final mixture was carefully poured into a clean Petri dish and kept at four degrees Celsius.

### 2.4. Characterization of Material

#### 2.4.1. Fourier-Transform Infrared (FTIR) Spectroscopy Analysis

The success of Gel and HA crosslinking with BDDE was confirmed by Fourier-transform infrared spectroscopy (Jasco, FT/IR-4200, Tokyo, Japan). 

#### 2.4.2. X-ray Diffraction (XRD) Analysis

The composition of the Gel/HA–HAP composite was characterized by X-ray diffraction (Rigaku, TTRAX 3, Tokyo, Japan). The parameters used were Tension 30 kV and Current 20 mA. The scanning degree was 2θ = 10°–80°, and the scanning rate was 10°/min.

#### 2.4.3. Degradation Profile

The degradation profile of the Gel/HA–HAP composite was tested by immersing the composite in PBS solution (pH = 7.4) at 37 °C. At various time points, the degraded gelatin solution was then measured at two hundred and thirty nanometers by an ELISA reader (Tecan, Sunrise, Australia).

#### 2.4.4. Swelling Ratio Test

To test the swelling ratio, the Gel/HA–HAP composite was incubated in phosphate-buffered saline (PBS, pH 7.4) at 37 °C. A known weight (W_dry_) of the composite was placed in PBS for various time lengths. The wet weight (W_swollen_) of the composite was determined immediately after the surface-adsorbed water was removed by filter paper. The swelling ratio was then calculated as follows:

(1) $$\mathrm{Swelling}\;\mathrm{ratio}\;\left(\%\right)=\frac{W_{\mathrm{swollen}}-W_{\mathrm{dry}}}{W_{\mathrm{dry}}}\times 100\%$$

#### 2.4.5. Insulin Release Profile

FITC–insulin was chosen as the model drug for SDF-1 due to the cost of SDF-1. Insulin has not only a similar shape, but also a similar molecular weight compared with SDF-1: six kilodaltons and eight kilodaltons, respectively. As a result, these two molecules may share similar release profiles. For the release profile test, the Gel/HA–HAP–FITC–insulin composite was immersed in PBS solution (pH = 7.4) at 37 °C. At various time points, the released FITC–insulin solution was measured at four hundred and eighty-eight nanometers by an ELISA reader.

### 2.5. In Vitro Studies

#### 2.5.1. Culture of Cells

Firstly, cbMSC-hTERT cells were cultured using α-minimum essential medium (MEM) supplemented with 1% of penicillin/streptomycin/Amphotericin B, 30 mg/L of hygromycin, 4 μg/L of recombinant human basic fibroblast growth factor (rhbFGF), 1.0 mmole/L of sodium pyruvate, and 20% of fetal bovine serum (FBS). These cells were cultured in a humidified incubator at 37 °C in an atmosphere of 5% CO_2_ [30].

#### 2.5.2. Viability of Cells

For the viability tests of cells, 10^4^ cells per well of cbMSC-hTERT cells were cultured for 24 h on a plate with ninety-six wells. One hundred microliters of the material extracts (0.2 g material per mL media) was added into the wells and incubated for 24 h. One hundred microliters of WST-1 solution (Taraka, Shiga, Japan) was added into the wells and incubated for two hours. Different absorbance values were measured for each well at four hundred and fifty nanometers using an ELISA reader. 

#### 2.5.3. Mesenchymal Stem Cell (MSC) Recruitment Test

We added 10^6^ cbMSC-hTERT cells into each well in six-well plates and cultured them for 24 h. Half of the cells were removed with cell scrapers. Gel/HA–HAP and Gel/HA–HAP–SDF-1 composites were separately placed into six-well plates where cbMSC-hTERT cells were absent. The composite (1 cm length and 0.5 cm width) was placed in the left side of the well, and the distance between the cells and the composite was about 1 cm. Cell migration was observed at 0, 24, 48, and 72 h.

### 2.6. In Vivo Studies

#### 2.6.1. Animal Model

The animal model studies were performed strictly according to the protocol reviewed and approved by the Institutional Animal Care and Use Committee (IACUC) of the National Taiwan University College of Medicine and College of Public Health. Eighteen Wistar male rats around 300 g in weight were purchased commercially. The rats were kept in groups of three in different cages. They were provided adequate water and food. After arrival, these rats stayed in the animal house for one week to acclimate to the environment. During implantation, the selected rats were first anesthetized by isoflurane, and all the fur near right knee joint was removed thoroughly. A linear incision was made along right knee joint to expose the bony structure of the knee. A defect about 2.5 mm in diameter and 5 mm in depth was made on the femoral condyle using a trephine drill [31,32,33,34,35]. In the control group, bone defects were left alone without any implants. In the other two groups, Gel/HA–HAP and Gel/HA–HAP–SDF-1 composites were implanted inside the bone defects. After implantation, skin was carefully closed with 4-0 absorbable sutures. All the rats were gently positioned to recover under a warm lamp. After the operation, the rats were kept under intensive care until sacrifice. The rats were sacrificed one and two months after implantation, and whole femur bones containing the defects and implants were collected. 

#### 2.6.2. Second Harmonic Generation (SHG) Imaging

After the animals were sacrificed, one fresh femur specimen of each group was directly sent for SHG imaging. Using two-photon excitation microscopy, SHG signals of the rat femur bone surface were recorded. SHG is an optical process in which materials with characteristic crystalline structures, such as type I collagen, convert two incident photons into a single photon with double energy. With an incident ultrafast laser wavelength of 890 nm, the microscope detects SHG signals in the 445 ± 10 nm window generated by type I collagen both from bone and soft tissue. Meanwhile, the microscope also detects autofluorescence signals of the specimen in the 500–530 nm range.

#### 2.6.3. Micro-CT Imaging 

All extracted tissue samples were kept in formaldehyde (10%) for seven days and then transferred into ethanol (95%) for micro-CT imaging. The condyle samples were scanned using a micro-CT (Bruker, Skyscan 1076, Billerica, MA, USA). Collected micro-CT images were then analyzed by computer to evaluate the bone healing process and residual implants. The degree of bone regeneration can be presented by calculating the bone-volume–tissue-volume ratio around the bone defect area. Software such as DataViewer was used to create two-dimensional (2D) images, while CTAn was used to generate three-dimensional (3D) images.

#### 2.6.4. Histological Imaging

The tissue samples were kept in formaldehyde (10%) for 7 days. After that, the samples were transferred into nitric acid (5%) for 3 days for decalcification. After being decalcified, the samples were sent for sequential alcohol dehydration from 70% to 100%. After dehydration, the samples were then put into xylene immersions. Finally, xylene was replaced by paraffin for infiltration. Before attaching to glass slides, paraffin blocks were cut into sections about five micrometers in size. H&E stains and MT stains were applied on different slides to visualize specific tissue and to evaluate new bone formation.

#### 2.6.5. Blood Tests

Cardiac punctures were performed with a needle (23G) under general anesthesia before sacrifice, and the collected blood sample was separated into two different types of collecting tubes. One collecting tube containing K2EDTA was sent for whole blood analysis, which includes red blood cell (RBC), hemoglobulin (HGB), hematocrit (HCT), mean corpuscular volume (MCV), mean corpuscular hemoglobin (MCH), mean corpuscular hemoglobin concentration (MCHC), white blood cell (WBC), neutrophil (NEUT), lymphocyte (LYMPH), monocyte (MONO), eosinophil (EO), and basophil (BASO). The other tube was centrifuged first to separate serum and blood cells. We then collected upper serum for biochemical analysis, including lactate dehydrogenase (LDH), alkaline phosphatase (ALKP), and calcium (Ca).

### 2.7. Statistics

ANOVA tests were performed for WST-1, micro-CT imaging and blood test results with MedCalc version 19.6.4.

## 3. Results

### 3.1. FTIR Analysis

**Figure 1:**
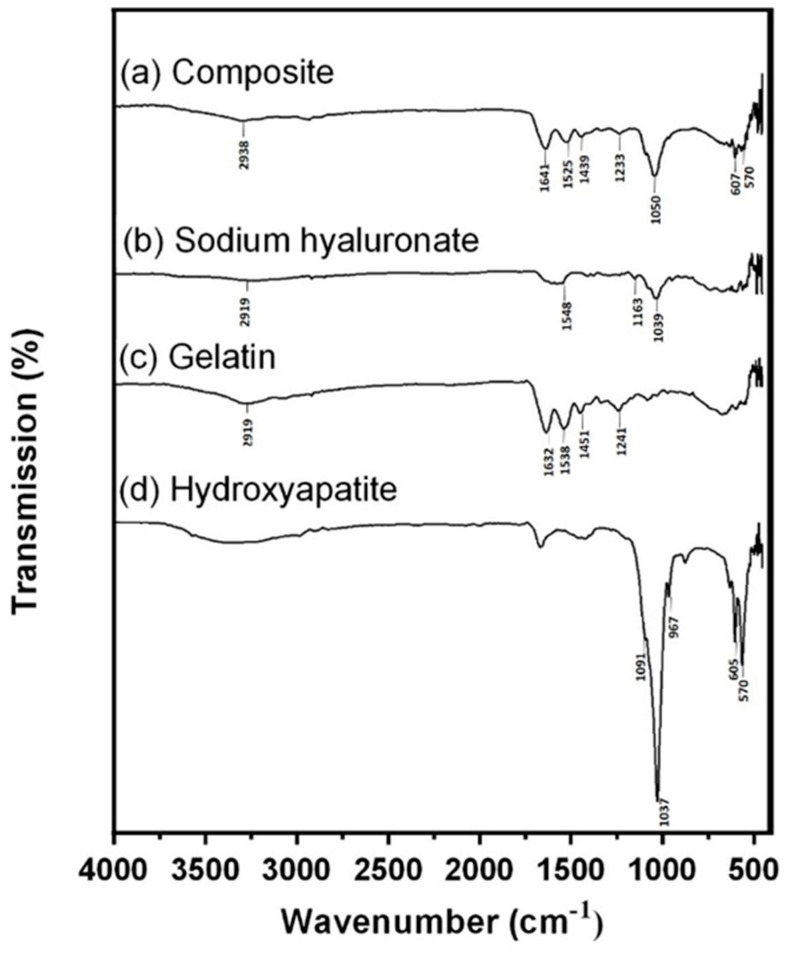
FTIR analysis. (**a**) Spectra of Gel/HA–HAP composite, (**b**) sodium hyaluronate, (**c**) gelatin, and (**d**) hydroxyapatite. The shallow absorbance band representing the C–H bond stretching of BDDE is located at 2958 cm^−1^, which indicates that hyaluronic acid and gelatin in the composite were crosslinked successfully.

FTIR spectra were used to confirm the crosslinking of the composite. HA and Gel were crosslinked by a crosslinker, BDDE, which formed many ether bonds. There were also many ether bonds in the backbone of HA. As a result, it was not likely to confirm the crosslinking process by using ether bond absorbance. On the FTIR spectrum of the Gel/HA–HAP composite (Figure 1a), a shallow absorbance band at 2958 cm^−1^ represented the stretching of the C–H bond on BDDE, indicating that the composite was crosslinked successfully. For the spectra of the composite, HA (Figure 1b), and Gel (Figure 1c), the absorbance bands of amide I C=O bond were at 1637–1639 cm^−1^, whereas 1520, 1542, and 1537 cm^−1^ bands revealed the absorbances of amide II N–H and C–N bonds. In both spectra of the composite and Gel, the absorbance bands of the N–H and amide III C–N bonds were noticed at 1235 cm^−1^. As shown in the spectra of the composite and HA, the absorbance bands of ether C–O–C bonds were between 1151 and 1159 cm^−1^. For the spectrum of HA, the absorbance band at 1030 cm^−1^ indicated an alcohol group C–OH bond. As for the spectrum of HAP (Figure 1d), the absorbance bands of P–O bonds were observed at 1091, 1030, 963, 631, 601, and 564 cm^−1^.

### 3.2. X-ray Diffraction Analysis

**Figure 2:**
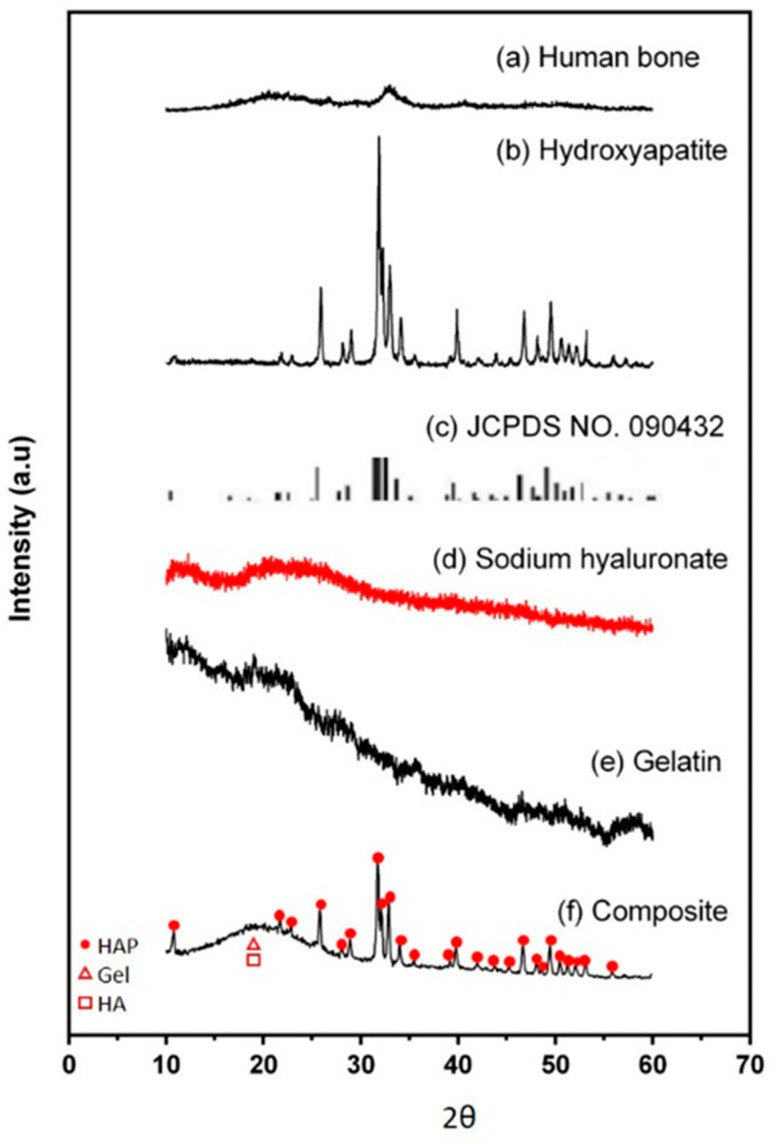
XRD analysis. (**a**) Human bone, (**b**) hydroxyapatite, (**c**) JCPDS No. 090432, (**d**) sodium hyaluronate, (**e**) gelatin, and (**f**) Gel/HA–HAP composite. The red dots, triangles, and rectangles indicate the characteristic peaks of hydroxyapetites, gelatin, and sodium hyaluronate, respectively. The Gel/HA–HAP composite prepared in this study had a similar composition to human bone.

Figure 2 reveals the XRD pattern of the Gel/HA–HAP composite. Compared to the pattern of human bone (Figure 2a), the composite under study had a similar composition to human femur bone. From the consistency between the self-prepared hydroxyapatite pattern (Figure 2b) and the standard pattern (Figure 2c) from the Joint Committee on Powder Diffraction Standard (JCPDS), the structure of hydroxyapatite was noted to be affected by the crosslinking process. From the patterns of gelatin (Figure 2e), sodium hyaluronate (Figure 2d), and Gel/HA–HAP composite (Figure 2f), they all shared a characteristic broad peak at 2θ = ~20°, which indicates the crystalline structure in hyaluronic acid and the triple-helical crystalline structure in gelatin. 

### 3.3. Degradation Test

**Figure 3:**
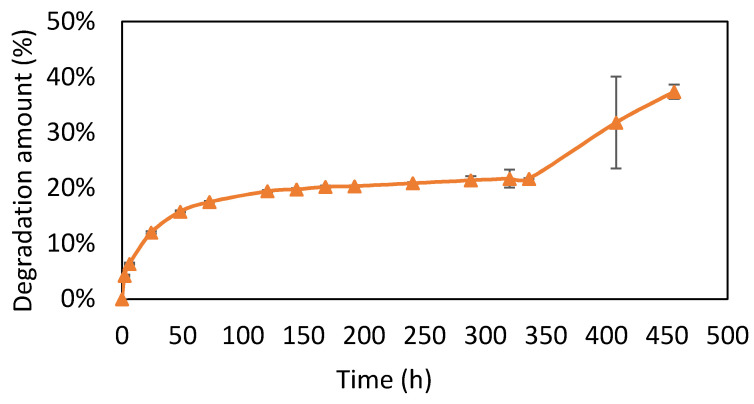
Degradation test (*n* = 6). Data are expressed as means ± SD. The Gel/HA–HAP composite had three stages of degradation.

The composite absorbed water and swelled, and degradation occurred. As shown in Figure 3, there were three stages of degradation. An initial fast burst can be noticed from 0 to 50 h, which is related to poorly crosslinked gelatin in the outer domain. Degradation in the phase of 50 to 350 h reached a plateau at about 20%, which indicates that residual gelatin was well crosslinked with HA. During the phase of 350 h onwards, the degradation of gelatin became faster again after HA breakdown.

### 3.4. Swelling Test

**Figure 4:**
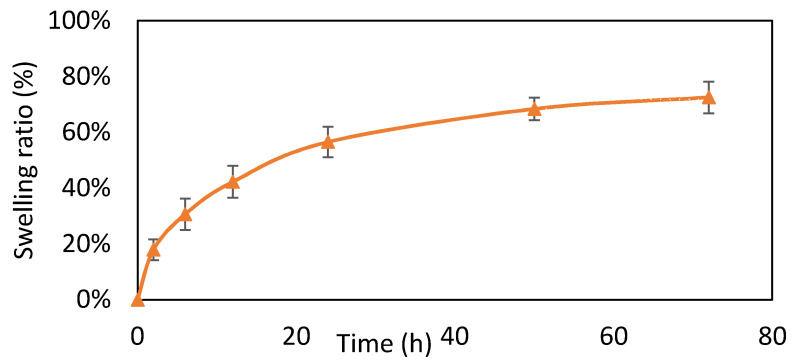
Swelling test (*n* = 6). Data are expressed as means ± standard deviation (SD). The composite showed two stages of swelling, including the first stage by HA and the second stage by Gel.

Swelling of the composite developed gradually after water absorption. After crosslinking, Gel absorbed less water. As a result, the swelling of the Gel/HA–HAP composite can be divided into two stages (Figure 4). In the first stage, the faster swelling rate of the composite was attributed to faster water absorption by HA. After HA absorbed most of water, the second stage of composite swelling was then attributed to Gel. 

### 3.5. Insulin Release Profile

**Figure 5:**
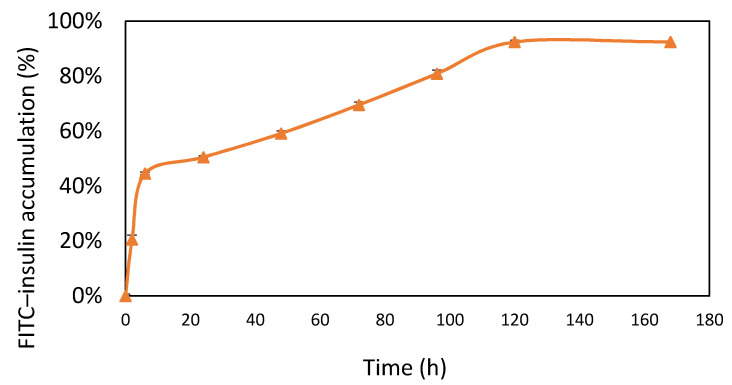
Insulin release profile (*n* = 6). Data are expressed as means. Gel/HA–HAP–FITC–insulin had two stages of release. The release rate was faster in the first stage, which was caused by the insulin molecules on the surface of the composite. The release rate was slower in the second stage, which was attributed to degradation of the composite itself.

Fluorescein isothiocyanate (FITC) had a maximum absorbance at 488 nm. As shown in Figure 5, the release of insulin was divided into two stages. Insulin was not crosslinked with HA and Gel; therefore, the first-stage release may be caused by insulin released from the surface of the Gel/HA–HAP–FITC–insulin composite. The second stage of insulin release was relatively slow and stable, and it lasted for about 5 days. This may have resulted from the insulin released from inside after the degradation of composite itself. Previous studies have shown that there is a binding domain for SDF-1 on collagen, whereas Gel is denatured collagen and HA is a polysaccharide. Therefore, it has been suggested that Gel has a higher affinity to insulin than HA [36,37,38]. The effective release was about 90%, and the course of the release of insulin lasted for about 5 days.

### 3.6. Viability of Cells

**Figure 6:**
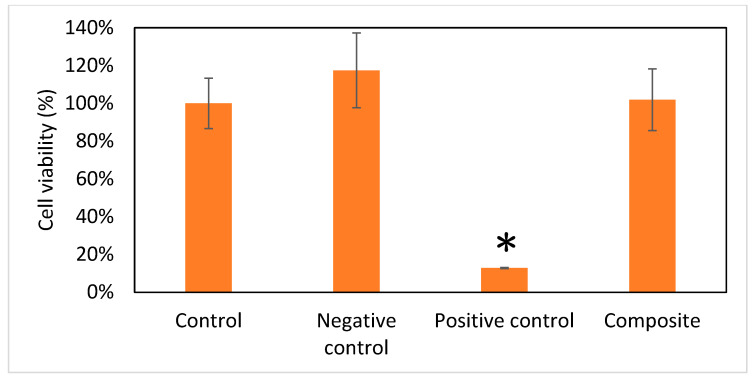
Viability of cells (*n* = 6). Data are expressed as means ± standard deviation (SD). Full cell viability (100%) was defined by the control group. Compared with the other three groups, the Gel/HA–HAP composite showed no cell toxicity. * *p* < 0.001.

WST-1 assays determined the cell viability and proliferation. Our target cbMSC-hTERT cells were treated with different media extracts as different groups. In the positive control group, 0.2 g/mL of zinc diethyldithiocarbamate was added; in negative control group, 0.2 g/mL of aluminum oxide was administered. Accordingly, 0.2 g/mL of Gel/HA–HAP composite was added as composite group. A 100% cell viability was defined for the untreated control group. From Figure 6, the growth of cbMSC-hTERT cells was not interfered with in the Gel/HA–HAP composite group or in the negative control group. In contrast, the viability of cbMSC-hTERT cells in the positive control group was obviously affected. Analysis of variance (ANOVA) revealed that only the positive control group showed a significant difference compared with the other three groups.

### 3.7. Mesenchymal Stem Cell Recruitment Test

**Figure 7:**
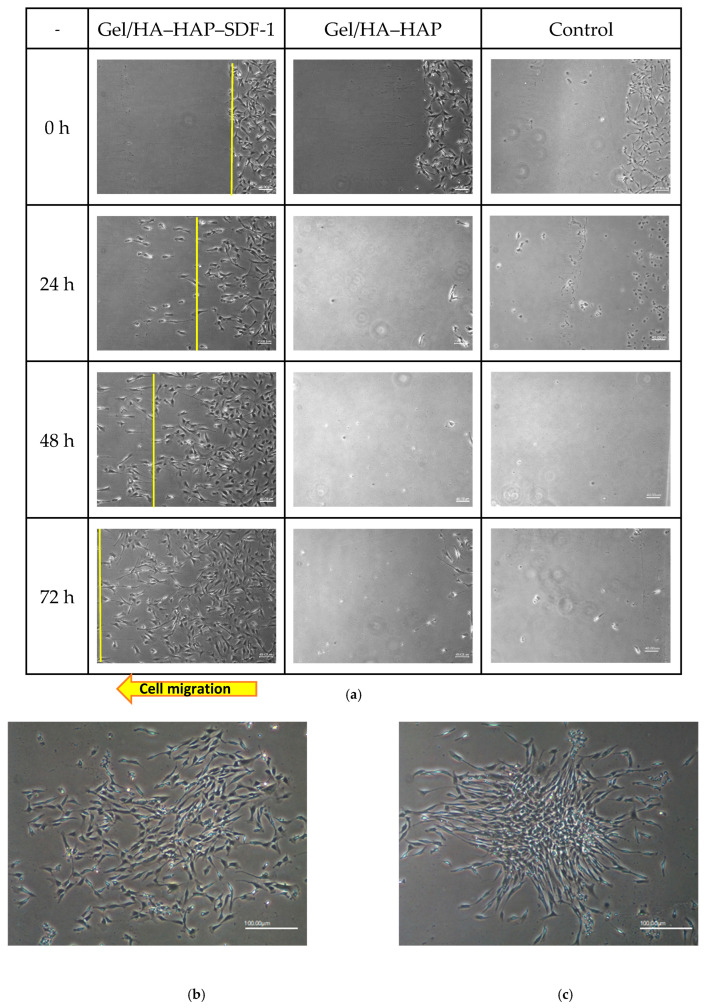

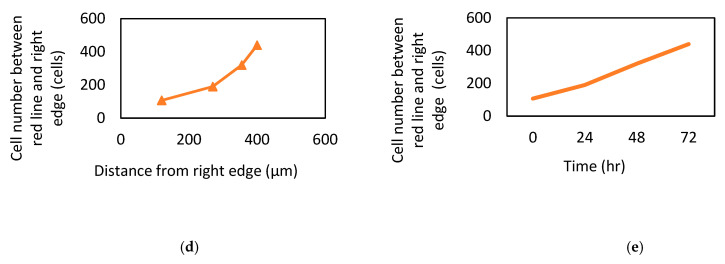
MSC recruitment tests and analysis. (**a**) Under 40× magnification, various time points of MSC patterns in the Gel/HA–HAP–SDF-1 group, Gel/HA–HAP group, and control group are presented from left to right. The front line of the MSCs is indicated by a yellow line. These images reveal that MSCs gathered together and colonized the control group and the Gel/HA–HAP group. MSCs in the Gel/HA–HAP–SDF-1 group migrated left, along the concentration gradient of SDF-1 released from the composite. After 24 h, MSCs in the control (**b**) and Gel/HA–HAP (**c**) groups gathered together without migrating toward one direction under 100× magnification. (**d**) Cell number of MSCs between the yellow line and right edge versus distance from the right edge in the Gel/HA–HAP–SDF-1 group. (**e**) Cell number of MSCs between the yellow line and right edge versus time in the Gel/HA–HAP–SDF-1 group.

MSC recruitment tests were used to determine the chemotaxis of SDF-1. From Figure 7a, at 0 h, the Gel/HA–HAP–SDF-1 group, Gel/HA–HAP group, and control group all had cbMSC-hTERT cells placed on the right side of well, and the material was placed on the left side. After 24 h, MSCs in the Gel/HA–HAP group and control group started to colonize because of their fibroblast-like nature (Figure 7b,c). In the Gel/HA–HAP–SDF-1 group, SDF-1 was slowly released and formed a concentration gradient. MSCs performed cell migration from right to left. The specific patterns in each group became more apparent after 48 and 72 h. We also performed analysis for MSC recruitment tests. In Figure 7d, as the distance increased from right edge, the cell number of MSCs increased. This indicates that MSCs did migrate toward the Gel/HA–HAP–SDF-1 composite, which also proved that SDF-1 was capable of attracting MSCs. From Figure 7e, as time increased, the cell number of MSCs between the yellow line and the right edge also increased. This revealed that MSCs moved toward the Gel/HA–HAP–SDF-1 composite as the time increased, which again confirmed the MSC recruitment ability of the composite. Furthermore, the nature of MSCs was maintained at the end of study, and there were no signs showing any impacts on MSCs made by SDF-1.

### 3.8. Second Harmonic Generation Imaging

**Figure 8:**
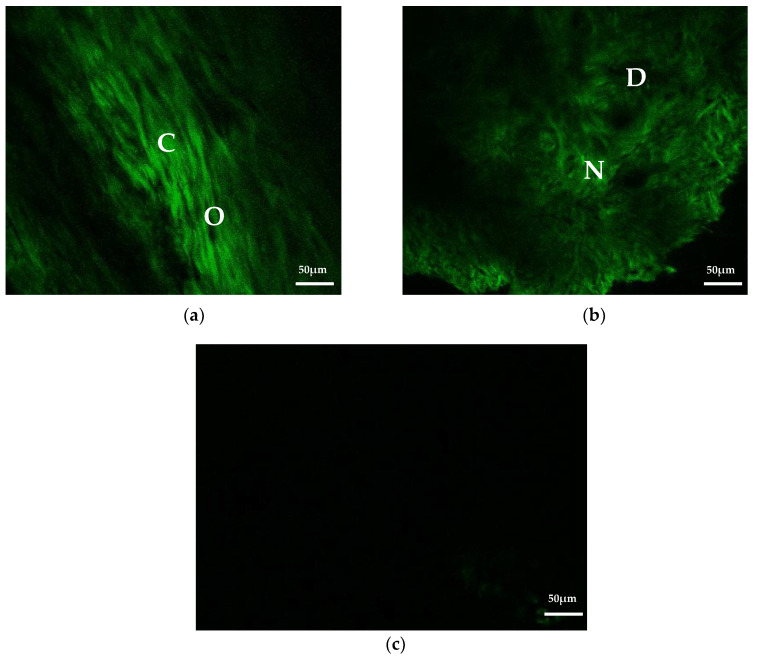
Second harmonic generation imaging. (**a**) Image of normal rat femur bone tissue. Well-aligned type I collagen fibers (C) are shown as green woven fibers, whereas the locations of osteocytes (O) can be observed at dark spindle-shaped areas. (**b**) The image of the drill hole that was filled with the Gel/HA–HAP composite for 1 month showed newly formed type I collagen fibers (N) around the composite, which evidenced new bone ingrowth near the bone defect area. Those dark areas (D) may indicate the residual HAP particles or those osteocytes that just migrated into this area. (**c**) Image of the Gel/HA–HAP composite itself before implantation shows no SHG signal at all.

After sacrifice, fresh femur specimens with or without composites were kept in normal saline and inspected with a two-photon microscope immediately for second harmonic generation (SHG) signals. Figure 8a displays a normal rat femur bone surface structure under SHG imaging. In normal bone structure, type I collagen fibers were presented as green woven fibers under SHG imaging, which were well-aligned. The location of osteocytes can also be noticed over those dark spindle-shaped areas. The image of a drill hole that was filled with the Gel/HA–HAP composite for 1 month can be seen in Figure 8b. There are some newly formed type I collagen fibers around the composite, which may indicate new bone ingrowth around the bone defect site. Compared with the collagen fibers in Figure 8a, these newly formed collagen fibers are much looser and poorly aligned. Additionally, there are some dark areas in Figure 8b, which may indicate the residual HAP particles or osteocytes that just migrated into this area. Figure 8c reveals the SHG image of the Gel/HA–HAP composite itself before implantation. The completely dark image indicates that there was no SHG signal generated from the composite, which was totally different from the image after implantation.

### 3.9. Micro-CT Imaging

**Figure 9:**
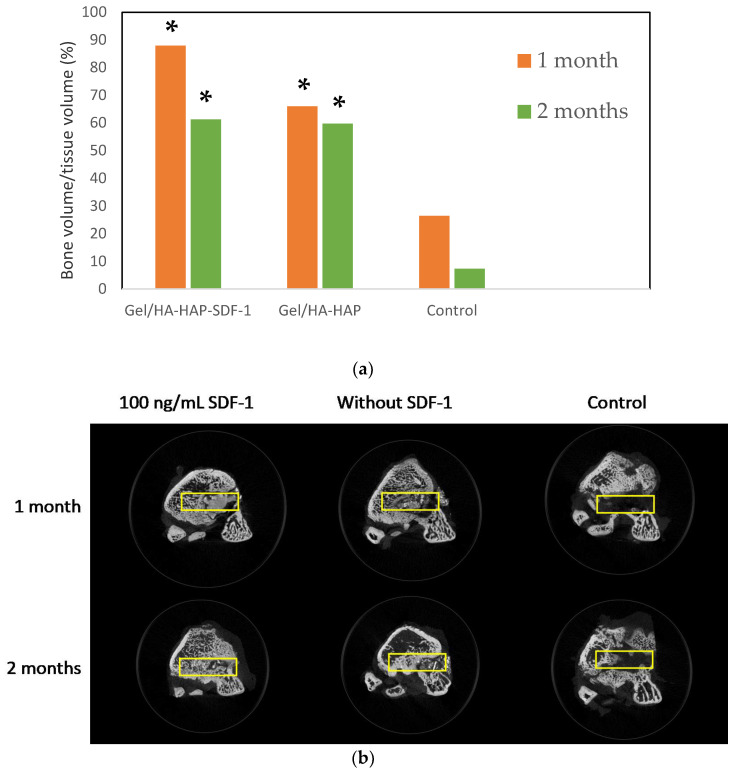
Micro-CT analysis and imaging. (**a**) Bone volume (BV)/tissue volume (TV) analysis over the bone defect sites of the scanned rat femur (*n* = 3). Compared with the control group, there was more new bone formation in both composite groups. * *p* < 0.001 (**b**) Micro-CT sections of femur specimens one month and two months after surgery. The bone defect sites are indicated with yellow rectangles.

All eighteen femur specimens were sent for micro-CT analysis after sacrifice. Figure 9a shows the extent of new bone formations in each bone defect site evaluated by the BV/TV ratio. The bone defect area was labeled under micro-CT imaging, as shown in Figure 9b. As mentioned above, the bone defect site created in the operation was a cylinder shape area about 2.5 mm in diameter and 5 mm in depth. By using CTan software, new bone formation in this defect area could be calculated and presented as the bone volume (BV)/tissue volume (TV) ratio. A higher BV/TV ratio indicates more new bone formation. As shown in Figure 9a, the BV/TV ratio after one month and two months in the Gel/HA–HAP–SDF-1 group were 86.35% and 59.25%, with standard deviations (SDs) of 5.83% and 9.27%, respectively. According to the micro-CT section of the Gel/HA–HAP–SDF-1 group in Figure 9b, some residual composite can still be seen after one month. Less composite and more bone-like material was observed after two months. As for the Gel/HA–HAP group, the BV/TV ratios after one month and two months in the Gel/HA–HAP group were 68.95% and 55.66%, with SDs of 10.05% and 7.75%, respectively. The micro-CT section of the Gel/HA–HAP group also showed some residual composite inside the bone defect area, and there was also some bone-like material in the defect area. As we can see, the composite remaining after two months was less than that after one month, and bone-like material was also more obvious after two months. For the control group, the BV/TV ratios after one month and two months in the control group were 26.46% and 7.29%, with SDs of 5.61% and 4.31%, respectively. The micro-CT sections show obvious defect sites either after one or two months, which indicates that there was only limited new bone formation in the control group.

The composites in the Gel/HA–HAP–SDF-1 and Gel/HA–HAP groups were gradually absorbed in vivo. According to the results of the swelling profile and degradation profile, it was suggested that swelling resulted in material degradation which then led to absorption after implantation. From Figure 9b, either one month or two months after surgery, both Gel/HA–HAP–SDF-1 and Gel/HA–HAP groups exhibited more bone-like material in the defect site compared with the control group. Compared with the BV/TV ratio of and the control group, both composite groups showed significantly better bone healing in either one month or two months. Furthermore, there was a slight trend of better bone healing in the Gel/HA–HAP–SDF-1 group compared with the Gel/HA–HAP group, which did not achieve statistical significance. 

### 3.10. Histological Imaging

**Figure 10:**
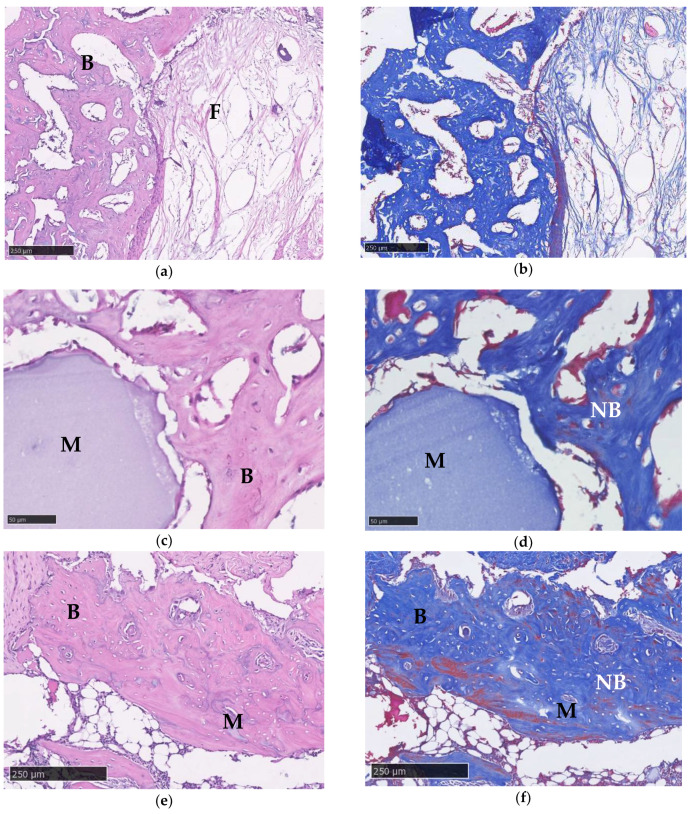

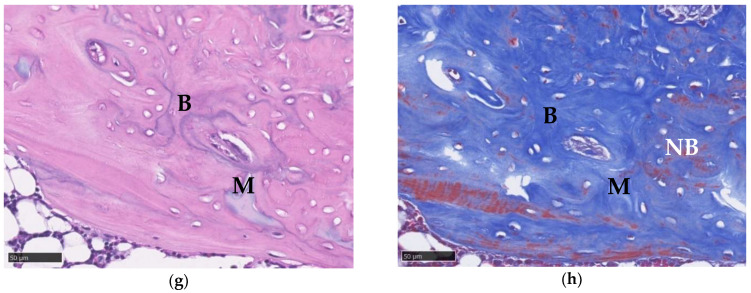
Histological imaging. (**a**) Control group under 10× magnification view showing a large bone defect filled with fibrotic tissue [F] next to a normal bone structure [B] under H&E. (**b**) Control group with MT staining under 10× magnification. (**c**) Gel/HA–HAP group under 40× magnification with H&E staining, residual material [M] can also be observed. (**d**) Gel/HA–HAP group under 40× magnification with MT staining. Blue stains represent collagen and bone matrix, whereas red stains show cytoplasm and osteoid. Possible new bone [NB] formation can be found near material. (**e**) Gel/HA–HAP–SDF-1 group with H&E staining under 10× magnification. Residual material was smaller and was well integrated into the normal bone structure. (**f**) Gel/HA–HAP–SDF-1 group with MT staining under 10× magnification. Some possible new bone formation was also noted. (**g**) Gel/HA–HAP–SDF-1 group with H&E staining under 40× magnification. (**h**) Gel/HA–HAP–SDF-1 group with MT staining under 40× magnification.

Hematoxylin and eosin (H&E) and Masson’s trichrome (MT) staining were used for histological imaging. For MT staining, the blue stains indicate collagen fibers and mature bone, whereas the red stains represent cytoplasm and new bone. In the control group, both H&E and MT staining showed obvious bone defects after 2 months (Figure 10a,b). In Gel/HA–HAP group, histology imaging revealed new bone formation under H&E and MT staining (Figure 10c,d). Large residual materials of composite can also be observed. Figure 10e,f reveal histology sections of the Gel/HA–HAP–SDF-1 group. The size of residual material seems to be smaller in the Gel/HA–HAP–SDF-1 group compared with the Gel/HA–HAP group.

### 3.11. Blood Tests

Blood sampling was performed via cardiac puncture under anesthesia before sacrifice. Blood samples were sent for whole blood tests and biochemistry tests, as mentioned above. The results of blood tests were compared with the normal range in the literature (Charles River Laboratories, 1982). ANOVA tests showed no significant differences among all three groups, both in biochemistry and whole blood analyses. The means and standard deviations of the whole blood tests and biochemistry tests after 1 and 2 months are shown in Table 1 and Table 2. As shown, all values of the whole blood tests in all groups were in the normal range. For the biochemistry test, Ca and LDH values of all groups were in normal ranges. ALKP values in control group were slightly higher than the normal range for two-month dates, which may be related to the induced bone defects. In summary, the results of blood tests suggested that the Gel/HA–HAP–SDF-1 composite prepared in this study showed no obvious systemic toxicity.

## 4. Discussion

In this study, Gel/HA–HAP–SDF-1 and Gel/HA–HAP composites used as bone void fillers showed promising results. Good biocompatibility and biodegradability were confirmed by in vitro tests. Micro-CT imaging showed a significant increase in bone volume/soft tissue volume ratio in both composite groups compared to the control group. Histology findings also revealed new bone formations in both composite groups.

Other studies have already proved that such kinds of HA–HAP composites can be used as bone void fillers in animal models. Subramaniam et al. used hydroxyapatite (HAP)–calcium sulfate (CS)–hyaluronic acid (HA) composites encapsulated with collagenase (Col) on alveolar bone defects in a rat model [39]. They showed that their HAP/CS/HA–Col composite could enhance new bone formation in the study. However, the material they designed requires 20 min of setting time after mixture, which is not suitable for most orthopedic surgeries. The composite designed in our study does not need time to set, which can save much operation time during surgery. Faruq et al. combined hyaluronic acid–gelatin with biphasic calcium phosphate (BCP) granules on femur bone defects in rabbit [40]. Their study also demonstrated promising bone regeneration in a rabbit model. In our study, particles of hydroxyapatite were directly integrated into hyaluronic acid and gelatin as a composite, and a femoral condylar bone defect model in rat was used for in vivo study. However, the BCP granules in their study were actually separated from hydrogels, which may still have the risk of dislodging from bone defect sites under repetitive compressive force. Despite the difference in study designs, a comparable result of bone regeneration enhancement was noted in our study. Furthermore, most of the blood test parameters were within normal ranges in our study, which may indicate that there was no detectable systemic toxicity caused by implanted materials. As a result, the application of Gel/HA–HAP composites in traumatic bone defects is feasible. 

From the clinical aspect, the applicability of bone substitutes or bone grafts are important to consider when dealing with bone defects in surgery [5,41]. During operation, it is important that a bone void filler should be able to implant into bone defect areas in a relatively short time without dislodgement. In the present study, the Gel/HA–HAP composite we created had moderate hardness and adequate viscosity. The composite can be easily separated into a suitable size and shaped by hand. Surgeons can easily manipulate and fix this composite into the bone defect area without difficulty. One the other hand, most implanted bone substitutes with less viscosity can be washed out easily during operation. Owing to the adequate viscosity, the composite in this study also exhibited optimal stability after implantation without dislodgement. In short, the Gel/HA–HAP seems to be suitable for clinical practice.

The definite role of SDF-1 related to bone regeneration remains largely unclear. SDF-1 is well known to recruit MSCs [25,26,42,43,44], which was also confirmed in our study in vitro. Mesenchymal stem cells did migrate toward the composite, clearly releasing SDF-1, as compared with the other two groups. On the other hand, SDF-1 showed no obvious effect on the proliferation and multipotency of MSCs according to previous studies [33,34]. Although the exact role of MSCs during bone regeneration is not yet fully understood, we hypothesize that the recruitment of MSCs may provide a certain degree of osteoinductivity in the bone healing process, which may need more delicate study to confirm [22,45,46]. Lauer et al. developed a 3D-printed polylactide (PLA) cylinder and functionalized it with SDF-1 for use in a rat femur bone defect. They found that the addition of SDF-1 resulted in osteoinductive effects [47]. In our study, the Gel/HA–HAP–SDF-1 composite we made showed similar results. Bone growth in the Gel/HA–HAP–SDF-1 group showed a slight trend toward faster bone growth compared with the Gel/HA–HAP group.

There are only few studies that have used SHG imaging of bone structures [48,49,50,51,52]. One key reason is due to the limited penetration depths by contemporary laser beams, which may hinder visualization of the complete bone structure. Pendleton et al. evaluated lamella sheets and collagen fiber integrity in intact cranial bone by using SHG imaging [52]. From their study, they observed a loss of resolution as they imaged up to 70 μm deep into bone. In our study, we could also only observe the surface layers of bone structures. Even though, by SHG signals released from type I collagen fibers in a normal bone matrix, we could still observe bone surface structure and new bone ingrowth on implanted materials. By further developing this kind of method with delicate study design, live imaging of bone growth via SHG signals may be possible, which would be a powerful tool in bone regeneration studies. 

In our previous study, Gel/HA crosslinked composites adding HAP and calcium sulfate (CS) with or without SDF-1 were developed for enhancing bone regeneration [53]. However, the rapid resorption of CS in vivo did cause some problem clinically, such as delayed bone union or continuous wound discharge [54,55]. In this study, we used HAP only with a Gel/HA copolymer in order to obtain a more stable composite. Comparing the swelling, degradation and insulin releasing profile, the Gel/HA–HAP composites did show better stability than the composites with added CS. As for the bone regeneration in the animal model, Gel/HA–HAP composites also showed comparable results. Gel/HA–HAP–SDF-1 composites even showed a little more bone regeneration compared with composites adding CS (81.74% vs. 74.05% and 59.25% vs. 54.52% BV/TV after one month and two months, respectively).

The limitations of this study include the small number of animal models. Additionally, the fast bone generation speed of rats resulted in prompt bone growth in all groups after only one month. As a result, shorter observation intervals may be needed for more detailed comparisons between different groups. Furthermore, we used human SDF-1 in the rat model in vivo study. Although murine and human SDF-1 are highly similar and can also act across species, the extent of MSC recruitment may still possibly be compromised when using human SDF-1 in rats [56].

## 5. Conclusions

In summary, for managing traumatic bone defects, the Gel/HA–HAP–SDF-1 composite could be a feasible material for use as bone void fillers. More studies may be needed to confirm the best dosage of SDF-1 in the composite and the mechanism of how SDF-1 enhances bone growth.

## Figures and Tables

**Table 1:** Blood test (one month).

Component	Gel/HA–HAP–SDF-1	Gel/HA–HAP	Control	Reference *
RBC (M/µL)	8.28 (0.5)	8.46 (0.32)	8.09 (0.37)	7.37–9.25
HGB (g/dL)	14.57 (0.35)	14.3 (0.45)	14.27 (0.45)	14.4–17.6
HCT (%)	42.93 (1.8)	43.33 (1.76)	42.8 (0.62)	36–46
MCV (fL)	51.87 (2)	51.23 (0.38)	53 (2.02)	47–52
MCH (pg)	17.6 (0.75)	16.9 (0.17)	17.67 (0.25)	17–21
MCHC (g/dL)	33.97 (0.61)	33 (0.26)	33.33 (0.81)	35–43
WBC (K/µL)	12.38 (2.31)	12.33 (1.57)	12.2 (3.43)	6.19–12.55
NEUT (%)	8.93 (7.24)	13.53 (3.31)	13.57 (2.03)	1–29
LYMPH (%)	82.8 (7.23)	78.4 (4.45)	79.43 (3.11)	70–99
MONO (%)	7.13 (0.85)	5.8 (2.01)	5.2 (1.5)	0–6
EO (%)	1.07 (0.9)	2.17 (1)	1.67 (0.68)	0–3
BASO (%)	0.07 (0.06)	0.1 (0)	0.13 (0.06)	0–2
ALKP (U/L)	191.33 (10.02)	162 (21.07)	184.33 (88.95)	39–216
Ca (mg/dL)	9.77 (0.45)	9.77 (0.49)	9.1 (0.3)	8–15
LDH (U/L)	585.67 (100.95)	659 (161.15)	609 (193.9)	300–700

* Charles River Laboratories, 1982. (*n* = 3).

**Table 2:** Blood test (two months).

Component	Gel/HA–HAP–SDF-1	Gel/HA–HAP	Control	Reference *
RBC (M/µL)	8.33 (0.49)	8.42 (0.23)	8.27 (0.32)	7.37–9.25
HGB (g/dL)	14.77 (0.65)	14.5 (0.53)	14.63 (0.67)	14.4–17.6
HCT (%)	43.67 (2.5)	43.4 (2.43)	43.67 (1.57)	36–46
MCV (fL)	52.43 (1.16)	51.53 (1.53)	52.8 (0.4)	47–52
MCH (pg)	17.73 (0.35)	17.23 (0.21)	17.7 (0.44)	17–21
MCHC (g/dL)	33.8 (0.46)	33.43 (0.71)	33.53 (1)	35–43
WBC (K/µL)	12.53 (2.16)	12.3 (1.69)	11.97 (1.61)	6.19–12.55
NEUT (%)	14.8 (6.15)	14.3 (1.39)	20.87 (11.82)	1–29
LYMPH (%)	76.6 (4.97)	79.4 (1.4)	72.03 (12.42)	70–99
MONO (%)	4.87 (0.45)	4.03 (0.58)	4.27 (0.15)	0–6
EO (%)	3.6 (1.42)	2.07 (0.72)	2.63 (0.51)	0–3
BASO (%)	0.13 (0.06)	0.2 (0.1)	0.2 (0.1)	0–2
ALKP (U/L)	173.67 (24.54)	193.33 (36.9)	218.67 (45.32)	39–216
Ca (mg/dL)	10.77 (0.42)	10.43 (0.21)	10.77 (0.31)	8–15
LDH (U/L)	625.33 (276.3)	414.67 (24.79)	526 (121.9)	300–700

* Charles River Laboratories, 1982. (*n* = 3).
