# Correction: Chang et al. Fabrication of Stromal Cell-Derived Factor-1 Contained in Gelatin/Hyaluronate Copolymer Mixed with Hydroxyapatite for Use in Traumatic Bone Defects. *Micromachines* 2021, *12*, 822

**DOI:** 10.3390/mi16010037

**Published:** 2024-12-30

**Authors:** Yun-Liang Chang, Chia-Ying Hsieh, Chao-Yuan Yeh, Chih-Hao Chang, Feng-Huei Lin

**Affiliations:** 1 Department of Biomedical Engineering, National Taiwan University, No. 1, Sec. 1, Jen-Ai Road, Taipei City 10051, Taiwan; knifex@gmail.com (Y.-L.C.); larissa19950718@gmail.com (C.-Y.H.); 2 Department of Orthopaedic Surgery, National Taiwan University Hospital, No. 7, Chung Shan South Road, Taipei City 10002, Taiwan; 3 Integrative Stem Cell Center, China Medical University, No. 2, Yude Road, Taichung City 40447, Taiwan; joeyeh@gmail.com

The authors wish to make a change to the published paper [1], as several mistakes were made.

In the original publication, there were mistakes in Table 1 as published. The blood test data have been updated. The corrected Table 1 is presented below.

In the original publication, there were mistakes in Table 2 as published. The blood test data have been updated. The corrected Table 2 is presented below.

In the original publication, there were graphical mistakes in Figure 1 as published. The corrected Figure 1 is presented below.

In the original publication, there were graphical mistakes in Figure 2 as published. The corrected Figure 2 is presented below.

In the original publication, there were graphical mistakes in Figure 6 as published. The corrected Figure 6 is presented below.

In the original publication, there were graphical mistakes in Figure 7 as published. The corrected Figure 7 is presented below.

There was an error in the original publication. A correction has been made to the Results, subsection Blood Tests. The corrected part is presented below:

Blood sampling was performed via cardiac puncture under anesthesia before sacrifice. Blood samples were sent for whole blood tests and biochemistry tests, as mentioned above. The results of blood tests were compared with the normal range in the literature (Charles River Laboratories, 1982). ANOVA tests showed no significant differences among all three groups, both in biochemistry and whole blood analyses. The means and standard deviations of the whole blood tests and biochemistry tests after 1 and 2 months are shown in Table 1 and Table 2. As shown, all values of the whole blood tests in all groups were in the normal range. For the biochemistry test, Ca and LDH values of all groups were in normal ranges. ALKP values in control group were slightly higher than the normal range for two-month dates, which may be related to the induced bone defects. In summary, the results of blood tests suggested that the Gel/HA–HAP–SDF-1 composite prepared in this study showed no obvious systemic toxicity.

The authors state that the scientific conclusions are unaffected. This corrections have been approved by the Academic Editor. The original publication has also been updated.

## Figures and Tables

**Figure 1:**
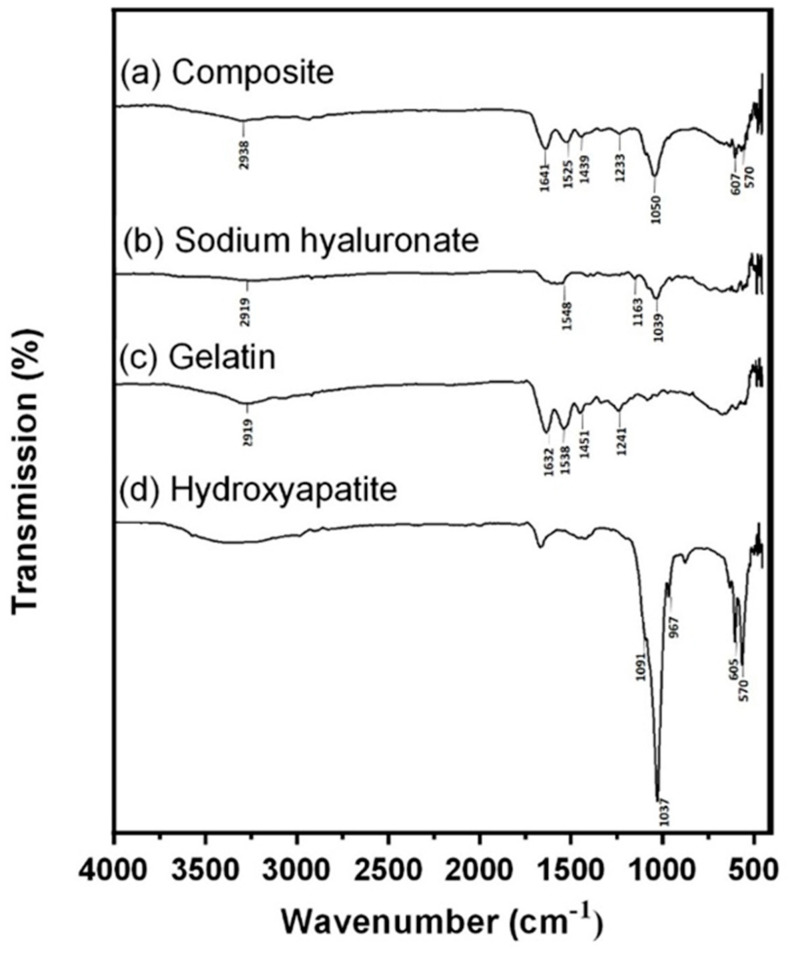
FTIR analysis. (**a**) Spectra of Gel/HA–HAP composite, (**b**) sodium hyaluronate, (**c**) gelatin, and (**d**) hydroxyapatite. The shallow absorbance band representing the C–H bond stretching of BDDE is located at 2958 cm^−1^, which indicates that hyaluronic acid and gelatin in the composite were crosslinked successfully.

**Figure 2:**
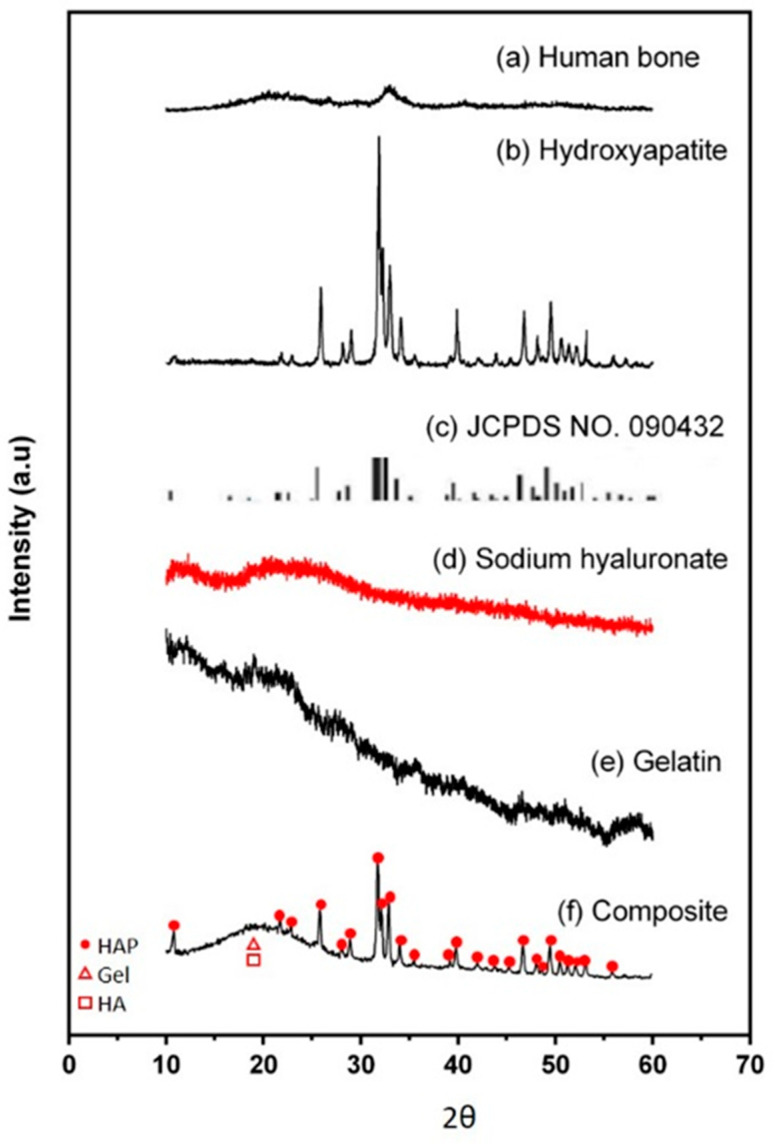
XRD analysis. (**a**) Human bone, (**b**) hydroxyapatite, (**c**) JCPDS No. 090432, (**d**) sodium hyaluronate, (**e**) gelatin, and (**f**) Gel/HA–HAP composite. The red dots, triangles, and rectangles indicate the characteristic peaks of hydroxyapetites, gelatin, and sodium hyaluronate, respectively. The Gel/HA–HAP composite prepared in this study had a similar composition to human bone.

**Figure 6:**
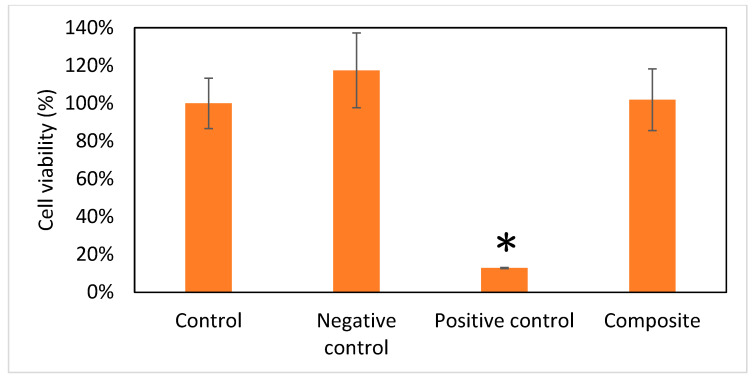
Viability of cells (*n* = 6). Data are expressed as means ± standard deviation (SD). Full cell viability (100%) was defined by the control group. Compared with the other three groups, the Gel/HA–HAP composite showed no cell toxicity. * *p* < 0.001.

**Figure 7:**
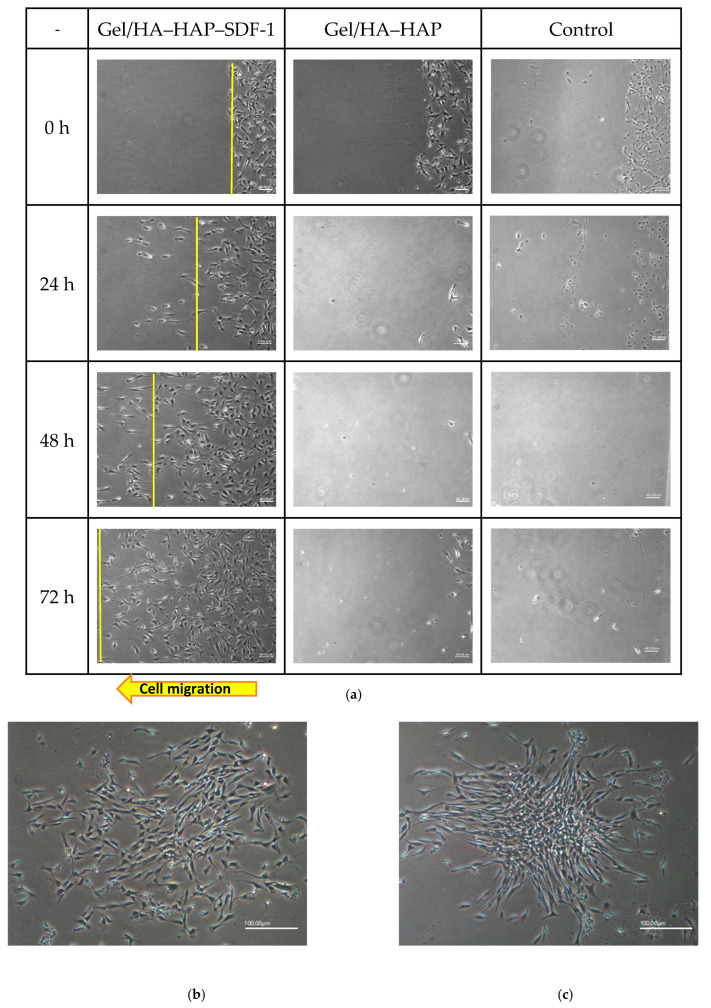

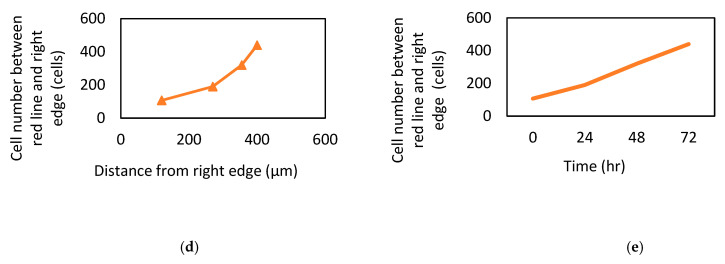
MSC recruitment tests and analysis. (**a**) Under 40× magnification, various time points of MSC patterns in the Gel/HA–HAP–SDF-1 group, Gel/HA–HAP group, and control group are presented from left to right. The front line of the MSCs is indicated by a yellow line. These images reveal that MSCs gathered together and colonized the control group and the Gel/HA–HAP group. MSCs in the Gel/HA–HAP–SDF-1 group migrated left, along the concentration gradient of SDF-1 released from the composite. After 24 h, MSCs in the control (**b**) and Gel/HA–HAP (**c**) groups gathered together without migrating toward one direction under 100× magnification. (**d**) Cell number of MSCs between the yellow line and right edge versus distance from the right edge in the Gel/HA–HAP–SDF-1 group. (**e**) Cell number of MSCs between the yellow line and right edge versus time in the Gel/HA–HAP–SDF-1 group.

**Table 1:** Blood test (one month).

Component	Gel/HA–HAP–SDF-1	Gel/HA–HAP	Control	Reference *
RBC (M/µL)	8.28 (0.5)	8.46 (0.32)	8.09 (0.37)	7.37–9.25
HGB (g/dL)	14.57 (0.35)	14.3 (0.45)	14.27 (0.45)	14.4–17.6
HCT (%)	42.93 (1.8)	43.33 (1.76)	42.8 (0.62)	36–46
MCV (fL)	51.87 (2)	51.23 (0.38)	53 (2.02)	47–52
MCH (pg)	17.6 (0.75)	16.9 (0.17)	17.67 (0.25)	17–21
MCHC (g/dL)	33.97 (0.61)	33 (0.26)	33.33 (0.81)	35–43
WBC (K/µL)	12.38 (2.31)	12.33 (1.57)	12.2 (3.43)	6.19–12.55
NEUT (%)	8.93 (7.24)	13.53 (3.31)	13.57 (2.03)	1–29
LYMPH (%)	82.8 (7.23)	78.4 (4.45)	79.43 (3.11)	70–99
MONO (%)	7.13 (0.85)	5.8 (2.01)	5.2 (1.5)	0–6
EO (%)	1.07 (0.9)	2.17 (1)	1.67 (0.68)	0–3
BASO (%)	0.07 (0.06)	0.1 (0)	0.13 (0.06)	0–2
ALKP (U/L)	191.33 (10.02)	162 (21.07)	184.33 (88.95)	39–216
Ca (mg/dL)	9.77 (0.45)	9.77 (0.49)	9.1 (0.3)	8–15
LDH (U/L)	585.67 (100.95)	659 (161.15)	609 (193.9)	300–700

* Charles River Laboratories, 1982. (*n* = 3).

**Table 2:** Blood test (two months).

Component	Gel/HA–HAP–SDF-1	Gel/HA–HAP	Control	Reference *
RBC (M/µL)	8.33 (0.49)	8.42 (0.23)	8.27 (0.32)	7.37–9.25
HGB (g/dL)	14.77 (0.65)	14.5 (0.53)	14.63 (0.67)	14.4–17.6
HCT (%)	43.67 (2.5)	43.4 (2.43)	43.67 (1.57)	36–46
MCV (fL)	52.43 (1.16)	51.53 (1.53)	52.8 (0.4)	47–52
MCH (pg)	17.73 (0.35)	17.23 (0.21)	17.7 (0.44)	17–21
MCHC (g/dL)	33.8 (0.46)	33.43 (0.71)	33.53 (1)	35–43
WBC (K/µL)	12.53 (2.16)	12.3 (1.69)	11.97 (1.61)	6.19–12.55
NEUT (%)	14.8 (6.15)	14.3 (1.39)	20.87 (11.82)	1–29
LYMPH (%)	76.6 (4.97)	79.4 (1.4)	72.03 (12.42)	70–99
MONO (%)	4.87 (0.45)	4.03 (0.58)	4.27 (0.15)	0–6
EO (%)	3.6 (1.42)	2.07 (0.72)	2.63 (0.51)	0–3
BASO (%)	0.13 (0.06)	0.2 (0.1)	0.2 (0.1)	0–2
ALKP (U/L)	173.67 (24.54)	193.33 (36.9)	218.67 (45.32)	39–216
Ca (mg/dL)	10.77 (0.42)	10.43 (0.21)	10.77 (0.31)	8–15
LDH (U/L)	625.33 (276.3)	414.67 (24.79)	526 (121.9)	300–700

* Charles River Laboratories, 1982. (*n* = 3).
